# Attitudes toward Physician-Nurse Collaboration in Pediatric Workers and Undergraduate Medical/Nursing Students

**DOI:** 10.1155/2015/846498

**Published:** 2015-07-26

**Authors:** Yong Wang, Yong-fang Liu, Hao Li, Tingyu Li

**Affiliations:** ^1^College of Economics & Business Administration, Chongqing University, Chongqing 400030, China; ^2^Department of Nutrition, Children's Hospital of Chongqing Medical University, Ministry of Education Key Laboratory of Child Development and Disorders, Chongqing 400014, China

## Abstract

The purpose of this study was to compare the attitudes of pediatric workers and undergraduate medical/nursing students toward collaboration. Attitude toward collaboration was measured using an adaptation of the Jefferson Scale of Attitude toward Physician-Nurse Collaboration. The 656 questionnaires were gathered from pediatrician, pediatric interns, and medical students (PIS) and pediatric nurses, nursing interns, and nursing students (NIS). Results showed a statistically significant difference in the total mean scores in attitudes towards collaboration with NIS scoring higher. Among the participants of PIS, the pediatricians obtained the highest mean scores, while, among the participants of NIS, the pediatric nurses got higher mean scores than nursing interns. It is desirable that medical and nurse schools should include interprofessional education in their curriculum to increase the understanding of the complementary roles of physicians and nurses and to encourage establishment of an interdependent relationship between them.

## 1. Introduction

It is suggested that interdisciplinary healthcare teamwork improves clinical outcomes as well as patient satisfaction and decreases institutional costs [1]. Correspondingly, collaborative relationships between physicians and nurses can positively influence clinical outcomes, such as patient care and patient death rates [2–4].

A recent integrated literature review [5] showed that ineffective physician-nurse collaboration has been shown to cause work dissatisfaction among physicians and nurses and compromised the quality of patient care. Another research [6] showed that positive physician-nurse collaboration relationships had a beneficial effect on the quality of drug use and the improvement of behavioral disturbance among a large number of nursing home residents. Because they are perceived to be important, physician-nurse professional collaborative relationships have long been a subject of often heated debate in medical and nursing literature.

Recently, numerous studies [7–9] were to investigate the attitudes toward physician-nurse collaboration in anesthesia, surgeons, operating room workers, and so on, while the study of collaboration in the pediatrician and pediatric nurses remains scant. When pediatricians and nurses work together, the nature of their interactions has the potential to influence the patient care they provide. Collaboration among pediatric healthcare workers has been advocated as a way to improve care delivery in an increasingly complex healthcare system [10]. In China, with limited healthcare resources and numerous healthcare demands, children's hospitals are always overcrowded with numerous patients, making the pediatrician and pediatric nurse under big pressure and high workload. Besides the government's effort to increase the number and quality of primary care institutions, positive physician-nurse collaboration is important for children's hospital to decrease medical accidents and improve performance, therefore to improve the quality of healthcare.

Collaboration is described as physicians and nurses cooperatively working together, sharing responsibilities for solving problems and making decisions to formulate and carry out plans for patient care [11]. Yet, in most researches, physicians and nurses view collaboration differently. Physicians have rated collaboration levels higher than their nursing counterparts, and nurses have valued collaboration more significantly positive than physicians [12–14]. Currently, it is reported that the healthcare system is experiencing a shortage of nurses, and the decline is partly due to unsatisfactory interprofessional relationships between physicians and nurses [15]. Because the interest to pursue nursing careers is in decline, due to the unsatisfactory physician-nurse relationships, there is a serious need to promote positive changes toward interprofessional collaboration between physicians and nurses [16]. Therefore, the question is, however, how to persuade physicians to be more willing to cooperate with other professionals in a healthcare centre. It is considered that the development of physician-nurse collaboration relationship was influenced by history, education, ethic, culture, gender, psychology, technology, and so on. The purpose of this study was to compare the attitudes of pediatric workers and undergraduate medical/nursing students towards collaboration. We also wanted to investigate whether such difference in attitudes towards collaboration could be related to gender or clinical practice experience.

## 2. Materials and Methods

### 2.1. Participants

The study was designed as a descriptive, comparative study with 6 naturally occurring groups (pediatrician, pediatric nurses, pediatric interns, nursing interns, medical students, and nursing students) to determine differences in attitudes toward collaboration. It was carried out from March 2013 to June 2014 in Chongqing, China. All pediatricians, pediatric nurses, pediatric interns, and nursing interns in the study were randomly selected from Children's Hospital of Chongqing Medical University. Then, all pediatrician and pediatric interns in the study distributed in all of the clinical departments, pediatric nurses and nursing interns were involved in direct patient care. Medical students and nursing students were randomly selected from Chongqing Medical University. PIS was defined, consisting of pediatrician, pediatric interns, and medical students; NIS was defined consisting of pediatric nurses, nursing interns, and nursing students.

### 2.2. Instrument

Data regarding attitudes toward collaboration were gathered using an adaptation of the Jefferson Scale of Attitudes toward Physician-Nurse Collaboration. The Jefferson Scale was originally developed to measure attitudes toward nurses and nursing services [17], which contained 20 items, and was slightly modified to investigate attitudes toward physician-nurse alliances. Five of the original 20 items did not have a significant factor coefficient on any of the extracted factors. These five items were deleted from the scale. The final version [18] of the Jefferson Scale is a survey consisting of 15 items answered on a 4-point Likert-type scale where the respondent could specify their stance from 1, which is equal to “strongly disagree,” to 4, which is equal to “strongly agree.” Thus, the attitude toward collaboration is reflected by the total score on the Jefferson Scale, ranging from 15 to 60, with higher scores indicating more positive attitudes. Results of an exploratory factor analysis provided support for the construct validity of this research tool. The prominent underlying factors of the Jefferson Scale of Attitudes toward Physician-Nurse Collaboration were identified as “shared education and teamwork” (7 items: 1, 3, 6, 9, 12, 14, and 15). Three other reliable factors were “caring versus curing” (3 items: 2, 4, and 7); “nurses' autonomy” (3 items: 5, 11, and 13); and “physician's dominance” (2 items: 8, 10). A higher factor score on the shared education and teamwork dimension indicates a greater orientation toward interdisciplinary education and interprofessional collaborations. A higher factor score on the caring versus curing dimension indicates a more positive view of nurses' contributions to psychosocial and educational aspects of patient care. A higher factor score on the nurses' autonomy dimension indicates more agreement with nurses' involvement in decisions on patient care and policies. A higher factor score on physician's dominance indicates rejecting a totally dominant role of physicians in aspects of patient care (items of this factor are reverse scored).

### 2.3. Procedures

The Jefferson Scale has been applied by international researchers to do comparison between physicians and nurses. The Chinese version of the Jefferson Scale was based on the version translated by Wang and Xu [19], which followed the back-translation process. (The Jefferson Scale was translated from English to Chinese by two of the coauthors. The Chinese version was translated back into English by two bilingual coworkers at the research department. A comparison between the translated and retranslated versions was made to assure the accuracy of the translation.) The survey was anonymous. Respondents did not sign their names, but they were asked to voluntarily provide information on their age, gender, professional status, and areas of specialization.

### 2.4. Statistical Analyses

Data were analyzed by SAS software (version 8.1). The significance level was set at 5%. This analysis was based on data from 656 participants. Descriptive statistics were compiled, Table 1. Comparisons of the total scores of the Jefferson Scale of Attitudes toward Physician-Nurse Collaboration were made on the raw scores. Then, data were presented as mean, median, and interquartile range (IQR). Moreover, we transformed the scores for the four factors (shared education and teamwork, caring versus curing, nurses' autonomy, and physician's dominance). A comparison of the independent groups was performed using the Mann-Whiney test, as well as *t*-text, for the analysis of data revealed a significant difference in attitude toward teamwork. Further analyses of the attitude score were compared by gender (male versus female), profession (physician versus nurse), and groups (different levels of clinical practice experiences) by using a three-way factorial analysis of variance design.

## 3. Results

### 3.1. Sample Characteristics

For the Jefferson Scale, a total of 656 respondents answered all the 15 questions. Then, all participants were divided into 6 groups by different levels of clinical practice experiences. Gender and median age for responding participants are summarized in Table 1. This sample included 118 pediatricians, 126 pediatric nurses, 98 pediatric interns, 118 nursing interns, 96 medical students, and 100 nursing students. The mean age of the above-mentioned participants in the region was 35 years, 30 years, 23 years, 21 years, 20 years, and 19 years, respectively, and 57% of the pediatricians were males. However, most participants were women in other groups.

### 3.2. Comparisons on Factor Scores

As reported in Table 2, a general pattern was observed in which the highest mean scores on the factor of “physician's dominance” were obtained by all participants, which was significantly different from each group. On the factors “shared education and teamwork” and “nurses' autonomy,” the nursing interns and nursing students obtained higher mean scores than pediatric intern and medical students, while, on the factor “caring versus curing,” medical students obtained significantly higher scores than those of nursing students.

The score for the attitudes towards collaboration was shown in Table 3. The mean score for PIS was 47.9 and the median was 47. For the NIS, the mean score was 48.33 and the median was 48. The analysis of data revealed a significant difference in attitude towards teamwork between PIS and NIS, while NIS demonstrated a more positive attitude towards teamwork than PIS (*χ* ^2^ = 3.88, *P* = 0.04). On the factors “shared education and teamwork” and “caring versus curing,” NIS obtained higher mean scores than PIS (mean scores 20.58 versus 19.26, 10.3 versus 9.72), while, on the factor “physician's dominance,” PIS obtained higher mean scores than NIS (*t* = 2.92, *P* = 0.03). Significant difference mean scores were not found between PIS and NIS on the factor “nurses' autonomy” (mean scores 10.11 versus 10.09). Moreover, among the participants of PIS, the pediatricians obtained the highest mean scores (*t* = 2.52, *P* = 0.03), and the medical students obtained the lowest mean scores, while, among participants of NIS, pediatric nurses got higher mean scores than nursing interns (*t* = 2.50, *P* = 0.03).

### 3.3. Three-Way Factorial Analysis of Variance Design

Because of the statistically significant interaction effects of participants by different levels of clinical practice experience, further analyses were performed to examine similarities and differences among the 6 study groups of participants. As reported in Table 4, the results did not indicate any significant difference in attitude scores between male and female in PIS and NIS. However, there was significant difference in attitude towards teamwork between PIS and NIS (profession: physicians versus nurses). We correlated the scores on the attitude scale with different levels of clinical experience for participants in the 6 study groups. Significant difference was found in the profession-group (different level of clinical practice experiences) variation.

## 4. Discussion

It is confirmed that physician-nurse collaboration is very important to decrease medical accidents and improve performance. Up to now, there are 223 million children younger than 14 years old in China (National Bureau of Statistics of China, 2011). Children's hospitals specialized in providing children healthcare services are crowded with patients. Therefore, child healthcare is getting more and more concerns in China. Valid teamwork assessment is imperative to optimize patient outcomes. In this study, the Jefferson Scale was used to assess the overall attitude toward physician-nurse collaboration in pediatric workers and undergraduate medical/nursing students in a children's hospital and a medical university in Chongqing, China. In summary, the results showed a statistically significant difference in the total mean scores in attitudes towards collaboration with NIS scoring higher. A higher total score has been interpreted to indicate a more positive attitude toward collaboration.

Comparative analysis of attitudes among pediatric workers and undergraduate medical/nursing students: research suggests [20] that many factors can strongly influence different aspects of the complex dynamics of the physician-nurse relationships, such as education, prescribed societal roles, cultural norms, and gender. In this study, all participants were divided into 6 groups by different levels of clinical practice experience. Then, analyses were performed to examine similarities and differences among 6 groups of participants: pediatrician versus pediatric nurses, pediatric interns versus nursing interns, and medical students versus nursing students. The results showed that the medical students and nurse students obtained the lowest mean attitude scores on the total, as well as on the factors of nurses' autonomy. On the factors of shared education and teamwork and nurses' autonomy, the nursing interns and nursing students had higher mean scores than pediatric interns and medical students. Among the participants of PIS, the pediatricians obtained the highest mean score, while medical students obtained the lowest mean scores and so do NIS. The findings are consistent with many other researches [5] that the factors of different levels of clinical practice experience contribute significantly to attitude toward physician-nurse collaboration. When the undergraduate medical students or nursing students study in school, their main job is to study the knowledge of medicine or nursing theory. They may not be aware that it is very important to decrease medical accidents and improve performance by well physician-nurse collaboration, because they have not started or are rarely dealing with patients. In a research [21] in Swedish universities, medical students also show a more negative attitude toward collaboration. In this study, years of work experience were associated with more positive attitudes toward collaboration. This may reflect a “generation-gap” effect, or it may be that attitude toward collaboration changes as professionals gain more experience. Research designed to explore these issues further could add to our understanding of collaboration. It is desirable that medical and nurse schools should include interprofessional education in their curriculum to increase the understanding of the complementary roles of physicians and nurses and to encourage the establishment of an interdependent relationship between them. There are new grants coming from Robert Wood Foundation and AMA being given to medical schools to develop programs that require team collaboration between medical, nursing, and pharmacy students starting in freshman year in the US. It is recommended that the faculty have preparation to demonstrate role modeling for collaboration.

Recently, many researches [5, 7] have concluded that nurses are more interested in physician-nurse collaboration than physicians. Nurses have been more involved in the associated research activities than their physician counterparts. This is consistent with this study, which revealed a significant difference in attitude towards teamwork between PIS and NIS. Moreover, on the factors of “shared education and teamwork” and “caring versus curing,” the NIS obtained higher scores than PIS. On the factor of “physician's dominance,” the PIS obtained significant higher scores than NIS, and so do pediatrician versus pediatric nurses, pediatric interns versus nursing interns, and medical students versus nursing students. The comparison of results is helpful to identify the potential reasons concerning what caused the mean score gaps between physicians and nurses. It is suggested that physicians and nurses shall learn from each other through continual education.

Some studies indicated that women expressed more positive attitudes toward physician-nurse collaboration than men. However, showing an influence of gender on collaboration scores in this study failed. A study in 4 countries also displayed that gender was not a powerful contributing factor to prescribe the professional roles [22]. It was said that the influence of gender has been hampered by continued disproportionate gender distribution between physicians and nurses. Future investigations into the role of gender in collaboration could consider taking advantage of the large number of pediatric workers and undergraduate medical/nursing students who are male.

## The Limitations of the Study

The validity and the reliability of the Jefferson Scale were originally tested on students with limited practice experience, which might limit its utilization with practicing pediatrician and pediatric nurse. Furthermore, the participants were selected from just one hospital and a medical university. The results may be typical rather than universal. Next, we will choose 3 or 4 children hospitals and medical universities to test the finding in a large scale.

## Figures and Tables

**Table 1:** Descriptive data stating gender and median age for responding participants.

Participants		Gender		No answer	Age median
		Male	Female		
Pediatrician	118 (18.0%)	57 (48.3%)	59 (50.0%)	2 (1.69%)	35
Pediatric nurses	126 (19.2%)	12 (9.5%)	113 (90%)	1 (0.8%)	30
Pediatric interns	98 (114.9%)	28 (28.6%)	70 (71.4%)	0	23
Nursing interns	118 (18.0%)	10 (8.5%)	108 (91.5%)	0	21
Medical students	96 (14.6%)	22 (22.9%)	74 (77.1%)	0	20
Nursing students	100 (15.2%)	2 (2.0%)	98 (98%)	0	19
Total	656	131 (20.0%)	522 (79.6%)	3 (0.4%)	

**Table 2:** Comparisons of participants on total scores and four factors of the Jefferson Scale of Attitudes toward Physician-Nurse Collaboration.

Participants	Factors				
	Shared education and teamwork	Nurses' autonomy	Caring versus curing	Physician's dominance	Total score
Pediatrician (*n* = 118)	22.8 ± 3.00	9.7 ± 1.24	10.03 ± 1.45	6.23 ± 1.14	48.68 ± 5.62
Pediatric nurses (*n* = 126)	23.34 ± 2.15	9.96 ± 1.25	9.85 ± 1.17	4.98 ± 1.22	48.13 ± 4.22
*P*	0.12	0.44	0.21	<0.0001	0.67
Pediatric interns (*n* = 98)	22.48 ± 2.83	9.36 ± 1.31	9.89 ± 1.43	5.57 ± 1.05	47.30 ± 5.41
Nursing interns (*n* = 118)	24.03 ± 2.90	10.05 ± 1.29	10.18 ± 1.16	4.84 ± 1.31	49.11 ± 4.83
*P*	<0.0001	<0.0001	0.10	<0.0001	0.0012
Medical students (*n* = 96)	22.61 ± 2.53	9.33 ± 1.21	10.02 ± 1.26	5.59 ± 1.19	47.26 ± 4.36
Nursing students (*n* = 100)	23.74 ± 2.57	9.95 ± 1.22	9.58 ± 1.17	4.41 ± 0.95	47.63 ± 4.00
*P*	0.0006	0.003	0.003	<0.0001	0.68
PIS (*n* = 312)	22.63 ± 2.80	9.47 ± 1.26	9.98 ± 1.35	5.83 ± 1.17	47.90 ± 5.21
NIS (*n* = 344)	23.69 ± 2.56	9.99 ± 1.25	9.98 ± 1.19	4.77 ± 1.20	48.33 ± 4.42
*P*	<0.0001	<0.0001	0.21	<0.0001	0.04

PIS consists of pediatrician, pediatric interns, and medical students; NIS consists of pediatric nurses, nursing interns, and nursing students.

**Table 3:** Mean and median for the attitudes towards collaboration, within PIS and NIS, as measured by the Jefferson Scale items and total score.

	PIS (*n* = 312)			NIS (*n* = 344)		
	Mean	Median	IQR	Mean	Median	IQR
(1) A nurse should be viewed as a collaborator and colleague with a physician rather than his/her assistant	3.36	3.00	1	3.56	4.00	1
(2) Nurses are qualified to assess and respond to psychological aspects of patients' needs	3.18	3.00	1	3.40	3.00	1
(3) During their education, medical and nursing students should be involved in teamwork in order to understand their respective roles	3.42	3.00	1	3.47	3.00	1
(4) Nurses should be involved in making policy decisions affecting their working conditions	3.22	3.00	1	3.52	4.00	1
(5) Nurse should be accountable to patients for the nursing care they provide	3.49	4.00	1	3.34	3.00	1
(6) There are many overlapping areas of responsibility between physicians and nurses	2.90	3.00	2	2.86	3.00	1
(7) Nurses have special expertise in patient education and psychological counselling	3.32	3.00	1	3.38	3.00	1
(8) Doctors should be dominant authority in all healthcare matters	2.95	3.00	1	2.29	2.00	1
(9) Imagine yourself in a situation where you work at a hospital, what do you then think about the following statement: physicians and nurses should contribute to decisions regarding the hospital discharge of patients	2.87	3.00	1	3.05	3.00	0
(10) The primary function of the nurse is to carry out the physician's orders	2.86	3.00	1	2.48	2.00	1
(11) Nurses should be involved in making policy decisions concerning the hospital support services upon which their work depends	3.06	3.00	0	3.08	3.00	0
(12) Nurses should also have responsibility for monitoring the effects of medical treatment	3.17	3.00	1	3.16	3.00	0
(13) Nurses should clarify a physician's order when they feel that it might have the potential for detrimental effects on the patient	3.56	4.00	1	3.67	4.00	1
(14) Physicians should be educated to establish collaborative relationships with nurses	3.29	3.00	1	3.57	4.00	1
(15) Interprofessional relationships between physicians and nurses should be included in their educational programmers	3.22	3.00	1	3.52	4.00	1
Jefferson total	47.90	47	8	48.33	48	6

IQR = interquartile range; PIS consists of pediatrician, pediatric interns, and medical students; NIS consists of pediatric nurses, nursing interns, and nursing students.

**Table 4:** Summary results of three-way analysis of variance.

Source of variation	*F*_(1,653)_	*P*
Main effects		
Profession (physicians versus nurses)	7.56	0.006
Gender (male versus female)	0.97	0.32
Two-way interactions		
Profession-gender	1.06	0.37
Profession-groups (different levels of clinical experiences)	2.4	0.03
Three-way interaction		
Profession-gender-groups (different levels of clinical experiences)	1.9	0.06
